# Premature changes in neuronal excitability account for hippocampal network impairment and autistic-like behavior in neonatal BTBR T+tf/J mice

**DOI:** 10.1038/srep31696

**Published:** 2016-08-16

**Authors:** Giada Cellot, Laura Maggi, Maria Amalia Di Castro, Myriam Catalano, Rosanna Migliore, Michele Migliore, Maria Luisa Scattoni, Gemma Calamandrei, Enrico Cherubini

**Affiliations:** 1 International School for Advanced Studies, via Bonomea 265, 34136 Trieste, Italy; 2 Department of Physiology and Pharmacology, Sapienza University, Rome, Italy; 3 Institute of Biophysics, National Research Council, 90146 Palermo, Italy; 4 Department of Cell Biology and Neurosciences, Istituto Superiore di Sanità, Rome, Italy; 5 European Brain Research Institute, via del Fosso di Fiorano 64, 00143 Rome, Italy

## Abstract

Coherent network oscillations (GDPs), generated in the immature hippocampus by the synergistic action of GABA and glutamate, both depolarizing and excitatory, play a key role in the construction of neuronal circuits. In particular, GDPs-associated calcium transients act as coincident detectors for enhancing synaptic efficacy at emerging GABAergic and glutamatergic synapses. Here, we show that, immediately after birth, in the CA3 hippocampal region of the BTBR T+tf/J mouse, an animal model of idiopathic autism, GDPs are severely impaired. This effect was associated with an increased GABAergic neurotransmission and a reduced neuronal excitability. In spite its depolarizing action on CA3 pyramidal cells (in single channel experiments *E*_*GABA*_ was positive *to E*_*m*_), GABA exerted at the network level an inhibitory effect as demonstrated by isoguvacine-induced reduction of neuronal firing. We implemented a computational model in which experimental findings could be interpreted as the result of two competing effects: a reduction of the intrinsic excitability of CA3 principal cells and a reduction of the shunting activity in GABAergic interneurons projecting to principal cells. It is therefore likely that premature changes in neuronal excitability within selective hippocampal circuits of BTBR mice lead to GDPs dysfunction and behavioral deficits reminiscent of those found in autistic patients.

Autism Spectrum Disorders (ASDs) comprise a complex and heterogeneous group of neuro-developmental disorders characterized by impaired social interactions, deficits in verbal and non-verbal communication, restricted interests and stereotyped behaviors^1^. Although the etiology is still unknown, ASDs share overlapping symptoms, suggesting common deficits in some neuro-developmental pathways. One of these involves GABA_A_-mediated neurotransmission, known to play a crucial role in synaptic tuning and neuronal wiring late in pre, early in postnatal life^2^,^3^. A GABAergic dysfunction may lead to an excitatory/inhibitory (E/I) unbalance in specific neuronal circuits thought to be involved in sensory, mnemonic, social, and emotional processes^4^,^5^,^6^.

Mouse models of ASDs have been instrumental for identifying the molecular and cellular mechanisms underlying these disorders and for developing new therapeutically promising tools. Alterations of GABAergic signaling common to ASD patients, have been detected in animal models of syndromic forms of autism^7^ and more recently in BTBR T+tf/j (BTBR) mice^8^, a well-recognized animal model of idiopathic autism^9^. BTBR mice, a natural occurring inbred strain of mice, whose genetic background is still under investigation^10^, exhibit altered social interactions, early communication deficits^11^,^12^,^13^,^14^,^15^,^16^, restricted pattern of interests^17^,^18^,^19^ and stereotyped behaviors including increased self-grooming and marble burying^11^,^18^.

Here, we investigated whether changes in GABAergic signaling may be detected in BTBR mice at an early stage of postnatal development. This information is crucial because neuro-developmental disorders such as ASDs should be diagnosed as early as possible, when an immediate therapeutic intervention may maximize potential benefits.

We focused on a primordial form of network synchronization, the so-called giant depolarizing potentials or GDPs, present in the hippocampus during the first week of postnatal life, generated by the synergistic action of GABA and glutamate both depolarizing and excitatory^3^,^20^,^21^. The depolarizing action of GABA leads to intracellular calcium rise through voltage-dependent calcium channels and/or NMDA receptors. GABA-mediated calcium signals regulate a variety of developmental processes from cell proliferation, migration, differentiation, synapse formation and neuronal wiring. In particular, calcium transients associated with GDPs are crucial for enhancing synaptic efficacy at emerging glutamatergic^22^ and GABAergic synapses^23^.

We found that BTBR mice exhibit from birth a reduced neuronal excitability within the CA3 hippocampal circuit, which leads to impairment of coherent network oscillations such as GDPs and persistent deficits in behavioral functions.

## Results

We measured the membrane capacitance (C_m_) and the membrane input resistance (R_in_) of principal cells in the CA3 region of the hippocampus in slices obtained from BTBR and C57BL/6J (B6) control mice between P3 and P6. Similar values of C_m_ were detected in the two mouse strains (60 ± 3 pF and 64 ± 3 pF in BTBR and in B6 mice, respectively; *p* = 0.4). In contrast, a significant (*p* = 0.045) reduction in R_in_ values was observed in BTBR mice (771 ± 52 MΩ; n = 55 cells/20 animals) as compared to B6 (947 ± 71 MΩ; n = 46 cells/20 animals).

### GDPs dysfunction in the hippocampus of neonatal BTBR mice

Since ASDs are developmental disorders, we firstly investigated whether BTBR mice exhibit alterations in coherent network activity such as giant depolarizing potentials (GDPs), generated within the hippocampus by the synergistic action of glutamate and GABA, both depolarizing and excitatory at this early developmental stage^2^,^3^,^20^.

**Figure 1:**
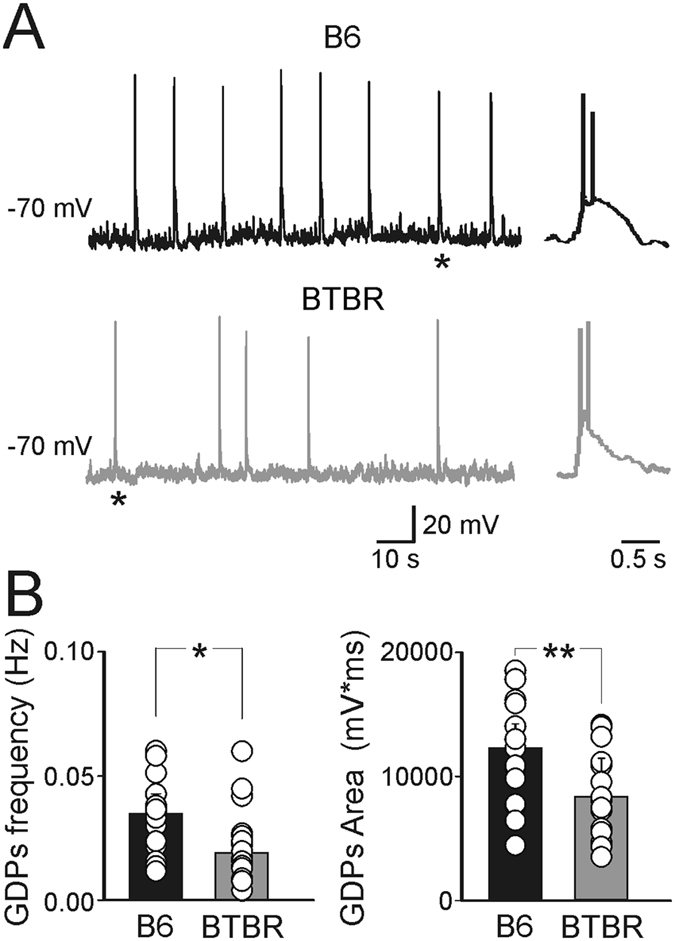
Impaired GDPs in neonatal BTBR mice. (**A**) GDPs recorded in hippocampal slices (at P3-P6) from B6 (black) and BTBR (grey) mice. GDPs marked with asterisks are shown on an expanded time scale on the right. (**B**) Each column represents the mean GDPs frequency and area from B6 (black, n = 24 cells/12 animals) and BTBR mice (grey, n = 24 cells/13 animals). In this and in the following Figures, open circles represent individual values. *p = 0.48; **p = 0.0049.

As shown in Fig. 1, on average a significant reduction in GDPs frequency (0.018 ± 0.002 Hz *vs* 0.036 ± 0.009 Hz; *p* = 0.048) and in the underlying area (8357 ± 646 mV^*^ms *vs* 12432 ± 1255 mV*ms; *p* = 0.0049) was observed in BTBR mice (n = 24 cells/13 animals) respect to B6 (n = 24 cells/12 animals). We measured also the firing rate and the number of spikes occurring during GDPs. No significant changes in these parameters were found in the two strains of mouse (during GDPs the firing rate was 2.76 ± 0.83 Hz and 3.64 ± 0.47 Hz, *p* = 0.34, while the number of spikes was 2.4 ± 0.38 and 2.2 ± 0.59, *p* = 0.81 in B6 and BTBR mice, respectively). In addition, no changes in overall firing rate was detected between the two genotypes (0.059 ± 0.02 Hz and 0.058 ± 0.01 Hz, *p* = 0.96, in B6 and BTBR mice, respectively). Although the ratio between the number of spikes occurring during GDPs and all spikes was reduced (79 ± 5% *vs* 89 ± 3%) in BTBR mice respect to B6, it did not reach a significant value (*p* = 0.11).

The early GDPs dysfunction may reflect alterations either in GABAergic, or glutamatergic signaling to CA3 principal cells. Therefore, in the following experiments spontaneous GABA_A_-mediated post synaptic currents (sGPSCs) were recorded from CA3 principal cells in both mouse strains in the presence of DNQX (20 μM) to block AMPA-mediated excitatory postsynaptic currents (sEPSCs).

### Increased GABAergic neurotransmission in the hippocampus of neonatal BTBR mice

**Figure 2:**
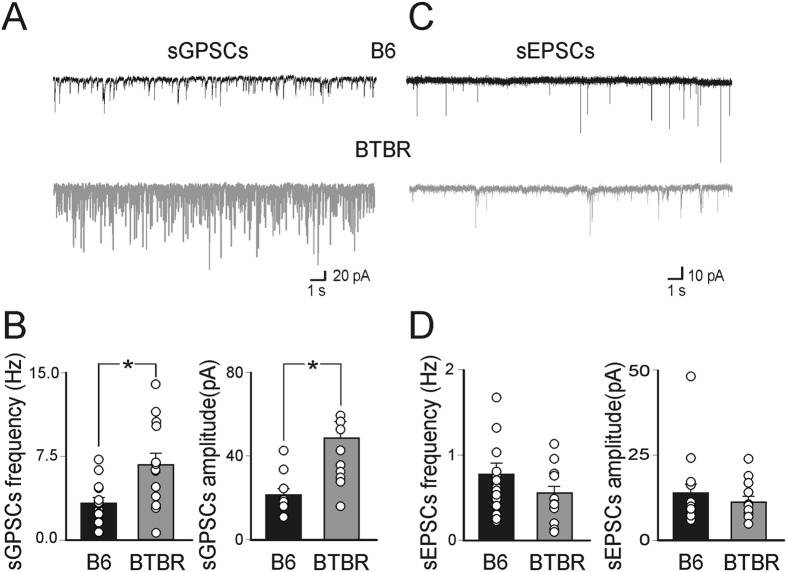
Altered GABAergic but not glutamatergic transmission in neonatal BTBR mice. (**A**) Sample traces of sGPSCs from B6 (black) and BTBR (grey) mice. (**B**) Each column represents the mean frequency and amplitude of sGPSCs in B6 (black, n = 13 cells/3 animals) and BTBR mice (grey, n = 14 cells/3 animals). *p < 0.05. (**C,D**), as for (**A,B**) but for spontaneous AMPA-mediated EPSCs (n = 15 cells/4 animals and 16 cells/3 animals for B6 and BTBR mice, respectively).

BTBR mice exhibited sGPSCs of higher amplitude that occurred more frequently with respect to B6 (Fig. 2A,B). On average, the amplitude of sGPSCs was 21 ± 3 pA and 49 ± 8 pA in B6 (n = 13 cells/ 3 animals) and BTBR mice (n = 14 cells/3 animals), respectively (*p* = 0.002). The frequency of sGPSCs was 3.4 ± 0.5 Hz and 6.7 ± 1 Hz in B6 and BTBR mice, respectively (*p* = 0.007). This effect was peculiar for the CA3 region, since recordings from CA1 principal cells in slices from age-matched animals revealed a reduction in frequency of sGPSCs (0.90 ± 0.16 Hz *vs* 1.57 ± 0.25 Hz; *p* = 0.03; data not shown) in BTBR mice (n = 18 cells/3 animals) respect to B6 (n = 17 cells/3 animals). Amplitude values were similar in both genotypes (44 ± 5 pA and 54 ± 5 pA, *p* = 0.13 in BTBR and B6 mice, respectively). These data are similar to those obtained by Han *et al*.^8^ in juvenile animals.

To understand whether changes in sGPSCs frequency and amplitude observed in CA3 principal cells depend on pre- or post-synaptic factors, we recorded miniature GABAergic events (mGPSCs) in the presence of TTX (1 μM) and DNQX (20 μM). As compared to B6 (n = 18 cells/3 animals), BTBR mice (n = 12 cells/4 animals) exhibited similar values of mGPSCs frequency (0.4 ± 0.07 Hz *vs* 0.4 ± 0.04 Hz, *p* = 0.88; data not shown), suggesting that modifications in the probability of GABA release and/or number of release sites do not contribute to GDPs alteration. However, they showed an increased amplitude value (24 ± 2 pA vs 18 ± 1 pA; *p* = 0.004) possibly related to an increased expression of postsynaptic GABA_A_ receptors, which in turn may contribute to alter shunting inhibition (see the computational model).

However, the similar values in decay kinetics (τ) of mGPSCs detected in the two strains of mice (τ values were 23 ± 1 ms and 21 ± 2 ms, in BTBR and B6 mice, respectively, *p* = 0.14), allow excluding changes in subunits composition of GABA_A_ receptors.

In contrast to sGPSCs, similar amplitude and frequency values of spontaneous AMPA-mediated EPSCs (recorded in the presence of bicuculline, 10 μM) were detected in both BTBR (n = 15 cells/4 animals) and B6 mice (n = 16 cells/3 animals). The amplitude of sEPSCs was 14 ± 3 pA and 11 ± 1 pA, (*p* = 0.36) while the frequency was 0.79 ± 0.13 Hz and 0.57 ± 0.08 Hz (*p* = 0.12) in BTBR and B6 animals, respectively (Fig. 2C,D). These results indicate that glutamatergic signaling to principal cells does not contribute to GDPs dysfunction.

### In spite its depolarizing action GABA inhibits the spontaneous firing of CA3 principal cells in BTBR mice

**Figure 3:**
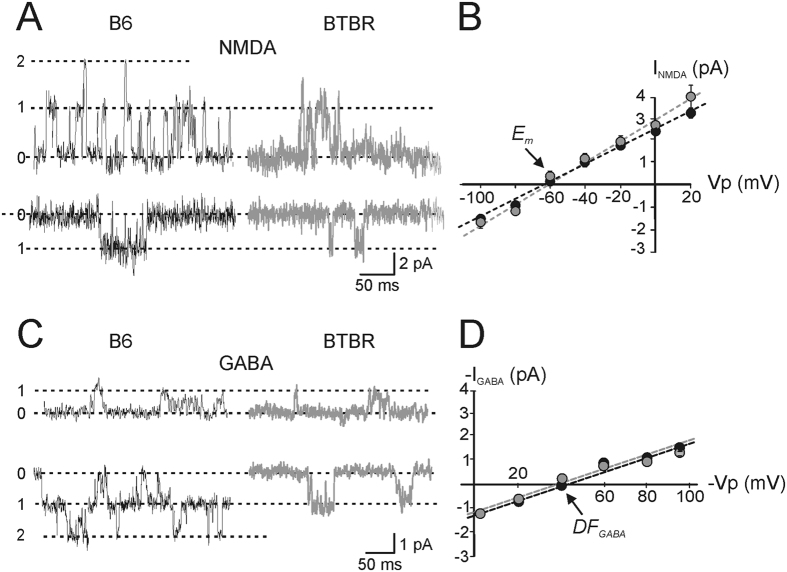
GABA depolarizes CA3 principal cells in neonatal BTBR as in B6 mice. (**A**) Samples of cell-attached recordings of NMDA-induced single-channel currents in CA3 principal cells from B6 (black) and BTBR (grey) mice. (**B**) Amplitudes of single NMDA currents are plotted *versus* pipette potentials (Vp) in B6 (black symbols, n = 13 cells/5 animals) and BTBR (grey symbols, n = 12 cells/7 animals) mice, respectively. The arrow in (**B**) indicates *E*_*m*_ values. (**C,D**) As in (**A,B**) but for GABA-induced single channel currents. Each point in (**B,D**) is the mean amplitude of NMDA (**B**) and GABA (**D**) openings obtained in 16 cells/3 animals for B6 and n = 16 cells/4 animals for BTBR mice at different values of *V*p. Arrow in (**D**) indicates DF_GABA_ values estimated from the reversal potential obtained by fitting I–V curves through data points.

The increased GABAergic neurotransmission observed in BTBR mice may be related to an increased GABAergic drive to CA3 principal cells. This may result from a depolarizing transmembrane chloride current with the equilibrium potential for GABA (*E*_GABA_) positive respect to the resting membrane potential (*E*_m_). As indicated in the methods (see ref. 24), single NMDA channels were used as voltage sensors to measure *E*_*m*_, while single GABA_A_ receptor channels were used to measure the driving force for somatic GABA_A_-mediated currents (*DF*_*GABA*_). Non-invasive cell-attached recordings allow estimating *E*_*GABA*_, being *E*_*GABA*_ = *DF*_*GABA*_ + *E*_*m*_. As shown in Fig. 3, similar values of *E*_*m*_ and *DF*_*GABA*_(estimated by the reversal of GABA-induced single channel currents) were detected in B6 and BTBR mice: *E*_*m*_ was −63.4 ± 1.5 mV and −61.5 ± 1.5 mV in B6 (n = 12 cells/7 animals) and in BTBR mice (n = 13 cells/5 animals; *p* = 0.48; arrow Fig. 3B), respectively; *DF*_*GABA*_ was 43.7 ± 2.5 mV and 42.5 ± 3.4 mV in B6 (n = 16 cells/3 animals) and BTBR mice (n = 16 cells/4 animals), respectively (arrow Fig. 3D; *p* = 0.79). *E*_*GABA*_ was −19.8 mV and −19 mV in B6 and in BTBR mice, respectively. Similar values of GABA- (~27 and 28 pS) and NMDA-induced single channel conductance (~40 and 41 pS) were detected in B6 and BTBR mice, respectively. These data indicate that GABA exerts a similar depolarizing action on CA3 principal cells in both B6 and BTBR mice.

**Figure 4:**
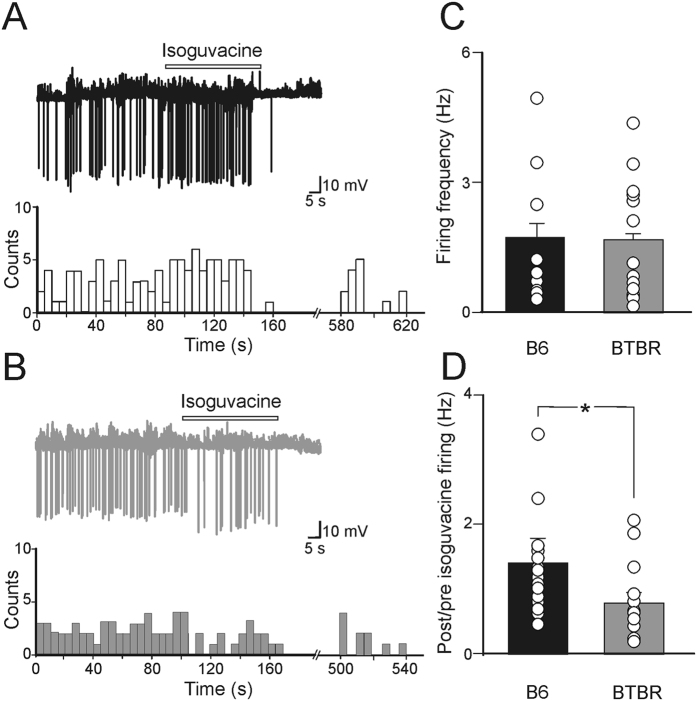
Isoguvacine reduces the firing of CA3 principal cells in neonatal BTBR mice. (**A**) Top trace: cell-attached recording of neuronal firing in the absence and in the presence of isoguvacine (10 μM, bar), a specific GABA_A_ receptor agonist, in a B6 mouse (black). Below, the histograms showing changes in firing rate during isoguvacine application (bin width: 5 s). (**B**) as in (**A**) but for BTBR mice. (**C**) Each column represents the mean firing frequency detected in the absence of isoguvacine in B6 (black, n = 16 cells/5 animals) and in BTBR mice (grey, n = 17 cells/7 animals). (**D**) Each column represents the ratio between the firing rate measured before and after isoguvacine in B6 (black, n = 16 cells/5 animals) and in BTBR mice (grey, n = 17 cells/7 animals). *p < 0.05.

We next evaluated whether, at the network level, the depolarizing action of GABA was associated with neuronal excitation. Therefore, in the following experiments, the effects of the specific GABA_A_ agonist isoguvacine was tested on the spontaneous firing of CA3 principal cells in both BTBR and B6 mice in cell-attached recordings that affect neither the membrane potential nor the [Cl^-^]i. Spontaneous firing occurred at 1.74 ± 0.6 Hz and 1.69 ± 0.31 Hz in B6 (n = 16 cells/5 animals) and in BTBR mice (n = 17 cells/7 animals), respectively (*p* = 0.94: Fig. 4C). While in B6 animals, isoguvacine (applied in the bath at the concentration of 10 μM) did not modify the firing frequency (the post/pre isoguvacine firing ratio was 1.62 ± 0.53, *p* = 0.88), in BTBR mice this drug reduced it in a statistically significant manner (the post/pre isoguvacine firing ratio was 0.87 ± 0.2, *p* = 0.035; Fig. 4B). The difference between the firing frequency (post *versus* pre application of isoguvacine) in B6 and in BTBR mice was statistically significant (*p* = 0.048; Fig. 4D). Hence, in BTBR mice, GABA inhibits newborn CA3 principal neurons probably by modifying, at the network level, the shunting inhibition.

Additional experiments were performed to see whether GABA released from GABAergic interneurons was still able to reduce the firing of principal cells in adult (P33-P45) BTBR mice. Stimulation of GABAergic interneurons in *stratum radiatum*, in the presence of DNQX (10 μM) to block AMPA receptors, caused a transient but strong reduction of cell firing in 7/8 cells from 3 animals in B6 and in 5/6 cells from 3 animals in BTBR mice. The ratio between the post and pre stimulus firing was 0.34 ± 0.09 (*p* < 0.01) and 0.45 ± 0.14 (*p* = 0.014) in B6 and in BTBR mice respectively; the difference between the two genotypes was not significant (*p* = 0.52; Fig. S1). These data indicate that the inhibitory action of GABA persists in adult BTBR mice as in B6.

### Reduced cell excitability in neonatal BTBR mice

**Figure 5:**
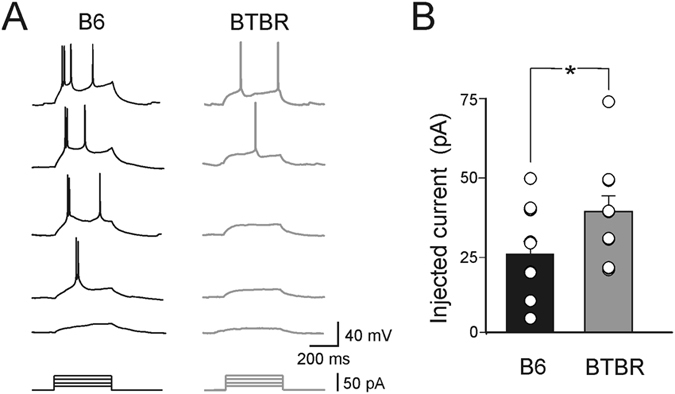
Reduced CA3 principal cells excitability in neonatal BTBR mice. (**A**) Sample traces showing the firing activity induced in CA3 principal cells by depolarizing current pulses of increasing amplitude (Δ 10 pA) from B6 (black) and from BTBR (grey) mice. Note the initial bursts firing in B6 but not in BTBR mice. (**B**) Each column represents the mean amount of current needed to induce firing activity in principal cells from B6 (black, n = 10 cells/3 animals) and BTBR (grey, n = 10 cells/3 animals) mice. *p < 0.05.

In a previous study from immature neocortical neurons^25^, the depolarizing and excitatory action of GABA was found to be associated with an enhancement of the intrinsic membrane excitability (*E*_*m*_ and spike threshold) of deep layer neurons. This combined action was thought to be responsible for spontaneous coherent network-driven oscillations. Therefore, in the following experiments to verify whether changes in cell excitability may contribute to the observed GDPs dysfunction we measured spike threshold in B6 and in BTBR mice. Although the firing threshold was exactly the same in both strains (−50 ± 1 mV and −50 ± 1 mV, *p* = 0.94), the amount of injected current needed to reach it was significantly higher in BTBR mice with respect to B6 (25.5 ± 4 pA and 39.5 ± 5 pA in B6, n = 10 cells/3 animals and in BTBR mice, respectively, n = 10 cells/3 animals; *p* = 0.04; Fig. 5).

It is worth noting that, in the majority of cases (70%), CA3 principal cells from B6 mice responded to long depolarizing current pulses with an initial burst of action potentials (Fig. 5A, black traces), whereas pyramidal neurons from BTBR mice responded with initial bursts only in 20% of cases (Fig. 5A grey traces). Changes in ionic conductances responsible for the spike after depolarization (which by re-depolarizing the neuron would generate additional spikes giving rise to bursts of action potentials) such as the persistent sodium current, low voltage-activated calcium currents, calcium-dependent potassium currents, I_M_, may account for the reduction in intrinsic neuronal excitability observed in BTBR mice^26^.

### Enhanced GABA_A_-mediated tonic inhibition in the hippocampus of neonatal BTBR mice

**Figure 6:**
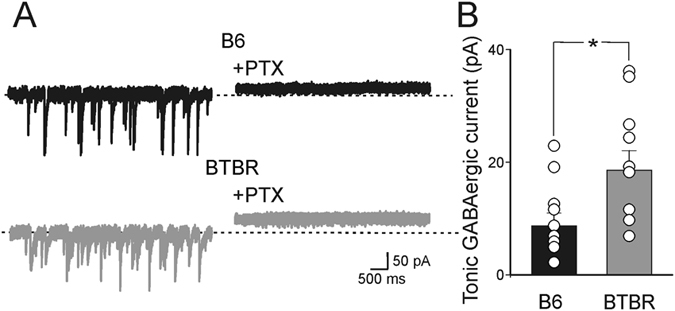
Tonic GABA_A_-mediated conductance in neonatal BTBR mice. (**A**) Representative traces of sGPSCs recorded from CA3 principal cells before (left) and after (right) application of picrotoxin (PTX, 100 μM) in hippocampal slices obtained from B6 (black) and BTBR (grey) mice. Please note the upward shift of the baseline after PTX application. (**B**) Each column represents the mean tonic GABA_A_-mediated conductance measured in B6 (black, n = 16 cells/3 animals) and BTBR mice (grey, n = 17 cells/4 animals). *p < 0.05.

The present data clearly show that, in spite of its depolarizing action on CA3 principal cells, in immature BTBR mice, GABA exerts an inhibitory effect on cell firing and correlated network activity, possibly by affecting at the network level shunting inhibition. This inhibitory action may be further boosted by tonic GABA_A_-mediated conductance, generated by the activation of extra-synaptic GABA_A_ receptors by spillover of GABA from adjacent synapses^27^. This conductance is known to be altered in several neuropsychiatric disorders including ASDs^28^. Therefore, in the following experiments we tested whether immature BTBR mice bear a tonic GABA_A_-mediated conductance that could contribute to alter GABAergic signaling. The tonic conductance was assessed by measuring in both B6 and BTBR mice the shift in the holding current induced by bath application of PTX, a GABA_A_ receptor channel blocker. PTX (100 μM) applied in the bath in the presence of DNQX (20 μM) caused a shift in the holding current larger in BTBR respect to B6 mice (19 ± 3 pA and 9 ± 2 pA, respectively; *p* = 0.037, n = 17 cells/4 animals and n = 16 cells/3 animals for BTBR and B6 mice, respectively; Fig. 6) suggesting that indeed an increased GABA_A_-mediated tonic conductance activated in BTBR mice by an enhancement of GABA release from GABAergic interneurons may contribute to alter GDPs.

### Modeling

**Figure 7:**
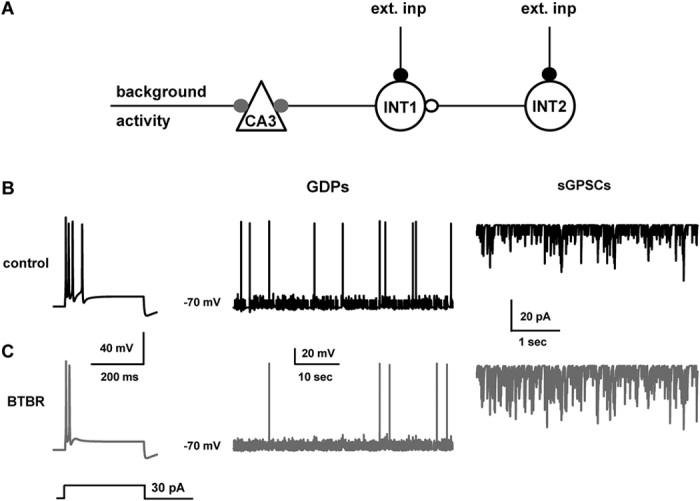
Computational model for alterations of GABAergic signaling in neonatal BTBR mice. (**A**) The microcircuit used for all simulations: two independent inputs activate excitatory synapses (ext.inp.) on interneurons. INT2 spikes activate a shunting synapse (open circle) on the soma of INT1, while INT1 spikes activate a depolarizing GABAergic synapse on the CA3 cell (closed grey circle). (**B**) (left) Somatic membrane potential of CA3 traces under control conditions during a 30 pA somatic current injection, (middle) typical pattern of spontaneous activity in the CA3 cell under control conditions, (right) typical time course of sGPSCs in the CA3 cell under somatic voltage clamp to −70 mV; (**C**), as in (**B**), but for BTBR.

Overall, the present data from neonatal BTBR mice clearly show that CA3 pyramidal cells are less excitable in spite of their increased level of GABAergic activity. These results are puzzling, since GABA, at this developmental stage, exerts on principal cells a depolarizing action. We thus used a computational model to hypothesize possible mechanisms underlying these experimental findings. A simple circuit was implemented using one CA3 pyramidal cell and two identical interneurons, connected as schematically shown in Fig. 7. Each interneuron received independent excitatory inputs (black symbols in Fig. 7A). One of the two interneurons (INT1) targeted the principal cell with a depolarizing GABA-ergic synapse (reversal potential of −19 mV). The other one (INT2) was connected to INT1 with a GABAergic synapse (reversal potential of −60 mV). Note that the low reversal potential of the INT2->INT1 synapse acts as a shunt on INT1, since its activation will generate a current with a driving force toward the resting potential. It will oppose any membrane deflection from other depolarizing/hyperpolarizing current. In other words, it acts as an additional membrane leak current.

Under control conditions, a somatic current injection generated a strongly adapting train of a few spikes (Fig. 7B, left), whereas spontaneous network activity generated GDP-dependent spikes (Fig. 7B, middle) and a barrage of sGPSCs recorded in voltage clamp conditions in the soma of the recorded cells (Fig. 7B, right). Because of the experimental findings of a reduced GDP area under BTBR conditions, we also ran additional simulations (not shown) by reducing the GDP decay time, and thus the area, by up to 50%. The overall number of spikes did not change. This result can be understood by considering that the action potentials occur at the end of the raising phase of the GDP, which is not much affected by the decrease of the area. One possible hypothesis is that, in BTBR mice, there are two competing effects: a reduction in the intrinsic excitability of CA3 pyramidal neurons and a reduction of the shunting activity on INT1 (resulting in an increase in both amplitude and frequency of sGPSCs).

We reproduced these effects with a 36% increase of the *K*_*M*_ peak conductance (from 0.33 to 0.45 nS/μm^2^, Fig. 7C, left trace, compare with Fig. 7B), and with a 6-fold decrease (from 60 to 10 nS) of the peak synaptic conductance of INT2 on INT2 (see methods for details). Overall, these changes resulted in a reduced network-driven activity (Fig. 7C, middle) and an increased spontaneous occurring GABA-ergic events (Fig. 7C, right), in qualitative agreement with the experimental findings. Note that, as in the experiments, the difference in the spontaneous spiking behavior was not statistically significant (Mann-Whitney rank sum test, *p* = 0.359). Taken together these results suggest that the increased GABA-ergic activity in BTBR mice is not sufficient to increase the firing of principal cells because of their reduced excitability.

## Discussion

The present data provide evidence that in the CA3 region of the hippocampus of BTBR pups, coherent network oscillations such as GDPs are severely impaired an effect probably related to a reduced network’s excitability. Although, at least in principal cells, single channel experiments have revealed a depolarizing action of GABA (*E*_*GABA*_ was positive respect to *E*_*m*_), this effect is paradoxically inhibitory as demonstrated by the ability of isoguvacine to reduce the firing rate of pyramidal cells, on cell attached experiments, that do not modify the intracellular chloride concentration in the recorded neurons. The unifying hypothesis, validated by our computational model, is that a reduced excitatory drive to principal cells and GABAergic interneurons contributes to disinhibit a set of GABAergic interneurons (that respect to principal cells have E_GABA_ closer to the resting membrane potential^29^), leading to an increased release of GABA from GABAergic interneurons. These modifications would result in alterations of the shunting inhibition with consequent impairment of GDPs expression and the excitatory/inhibitory (E/I) balance within the CA3 hippocampal circuit.

Early in postnatal life, an appropriate E/I balance is instrumental for the functional regulation of neuronal circuits^29^. Its disruption, during the period of synapse formation and consolidation, accounts for cognitive deficits associated with neuro-developmental disorders including mental retardation, schizophrenia, epilepsy and ASDs^4^,^5^,^6^,^30^,^31^,^32^,^33^,^34^,^35^. The E/I balance is maintained *via* highly regulated homeostatic mechanisms, involving ion channels, receptors, signaling pathways, and neurotransmitters^36^. Recent work on animal models of syndromic forms of autism suggests that the autistic-like behavior relies on changes of E/I neurotransmission in the brain^7^,^8^,^37^,^38^,^39^,^40^,^41^.

In a previous study from young-adult animals, a reduced level of spontaneous inhibitory transmission mediated by GABA_A_ receptors in the CA1 area of the hippocampus was detected. Interestingly, this effect and the associated autistic behavior could be rescued by low concentrations of positive allosteric benzodiazepine modulators of GABA_A_ receptors, suggesting that a GABAergic dysfunction is indeed at the origin of this disorder^8^. Similar results were obtained in the present experiments from immature CA1 principal cells, indicating that, alterations of GABAergic signaling are region-specific. As in CA1 hippocampal area, a weakened inhibitory circuit has been found also in the insular cortex of juvenile BTBR mice (and other monogenetic mouse models of autism) where it contributes to alter the capacity of integrating sensory input with emotional and cognitive processes, leading to social and communication deficits^42^.

Our data from neonatal CA3 hippocampal neurons, point to the impairment of early coherent oscillations (GDPs) as the possible primary cause of autistic deficits. The entire hippocampal network possesses the capacity to generate GDPs, but the CA3 area is particularly well equipped because of its extensive glutamatergic recurrent collaterals and spontaneous intrinsic bursts that can drive other neurons to fire^43^,^44^. In addition, here, GABAergic interneurons with expanded axonal arborizations operate as functional hubs able to synchronize a vast ensemble of cells^45^,^46^,^47^. This early synchronized activity, which may differ in its specific pattern among different brain regions, is crucial for synaptic wiring according to the Hebbian rule that “neurons that fire together wire together”^2^,^3^. In particular, GDPs, acting as coincident detector signals to enhance synaptic efficacy at emerging GABAergic^23^ and glutamatergic synapses^2^, exert a key role in the refinement of neuronal circuits before the development of more organized forms of synchronized activity such as theta and gamma rhythms^48^.

Why are GDPs altered in the CA3 area? In analogy with the synchronized activity generated in the disinhibited hippocampus, GDPs are thought to emerge when a sufficient number of cells fire and the excitability of the network attains a certain threshold within a restricted time window^49^. Although we did not characterize which sub-type of GABAergic interneuron is involved in GDPs dysfunction, parvalbumin-positive basket cells certainly contribute to the spontaneous action potential-dependent and -independent release of GABA^50^. In the hippocampus, parvalbumin-positive cells, already present at birth^46^, play a crucial role in coordinating the timing of neuronal activity, thus contributing to generate theta and gamma rhythms involved in high cognitive functions^51^,^52^,^53^. In addition, deficits in parvalbumin-positive interneurons have been detected in the cortex and in particular in the insula of syndromic and idiopathic animal models of autism, thus altering multisensory integration in this brain area^31^,^43^.

Whatever the type of inhibitory interneurons involved, the present experiments indicate that, in BTBR mice premature changes in neuronal excitability severely impairs coherent network activity and the E/I balance within the hippocampal circuit. This dysfunction may be implicated in the atypical behavioral phenotype reminiscent of that found in autistic children.

## Methods

### Animals

All experiments were performed in accordance with the Italian Animal Welfare legislation (D.L. 26/2014) that implemented the European Committee Council Directive (2010/63 EEC) and were approved by local veterinary authorities and by the ethical committee of SISSA and the Dept. of Physiology and Pharmacology, University of Roma, la Sapienza. All efforts were made to minimize animal suffering and to reduce the number of animal used.

BTBR and B6 mice were purchased from Jackson Laboratory (Maine USA). Age matched B6 mice were used as controls. Experiments were performed on both males and females. At least four mice from two different litters (in each strain) were used for a given experiment.

### Hippocampal slices

Transverse hippocampal slices (280 μm) were obtained from postnatal (P) day P3–P6 and P33-P45 animals, using a standard protocol^41^. Briefly, after being anesthetized with CO_2_, animals were decapitated. The brain was quickly removed from the skull and placed in ice-cold artificial CSF (ACSF) containing (in mM): NaCl 130, glucose 25, KCl 3.5, NaH_2_PO_4_ 1.2, NaHCO_3_ 25, CaCl_2_ 2, MgCl_2_ 1.3, saturated with 95% O2 and 5% CO2 (pH 7.3–7.4). Hippocampal slices were cut with a vibratome and stored at room temperature (22–24 °C) in a holding bath containing the same solution as above. After incubation for at least 1 h, an individual slice was transferred to a submerged recording chamber and continuously superfused at 33–34 °C with oxygenated ACSF at a rate of 3–4 ml min^−1^.

### Electrophysiology

A patch-clamp amplifier (multiclamp 700b, Axon Instruments, Sunnyvale, CA, USA) was used to record visually identified (with an upright microscope equipped with differential interference contrast optics and infrared video camera) CA3 and CA1 pyramidal neurons, using the whole cell patch-clamp technique in voltage and current clamp modes. Patch electrodes were pulled from borosilicate glass capillaries (Hingelberg, Malsfeld, Germany); they had a resistance of 4–7 MΩ when filled with intracellular solutions.

Spontaneous AMPA-mediated excitatory postsynaptic currents (sEPSCs) and GABA_A_-mediated postsynaptic currents (sGPSCs) were recorded from a holding potential of −70 mV in the presence of bicuculline (10 μM) and DNQX (20 μM), respectively. Miniature currents were recorded in the presence of TTX (1 μM) to block sodium currents and propagated action potentials. For glutamatergic currents we used an intracellular solution containing (in mM): K gluconate 120, KCl 20, HEPES 10, EGTA 10, MgCl_2_ 2 and Na_2_ATP 2 (pH 7.3 adding KOH). The same solution was used in current clamp experiments to record GDPs and action potentials threshold. In this set of experiments, 400 ms long lasting current steps of increasing amplitude (5, 10, 20, 30, 40, 50, 75, 100, 150 pA) were injected in pyramidal cells from an holding potential of −70 mV. For GABAergic currents we used an intracellular solution containing (in mM): CsCl 137, HEPES 10, BAPTA 11, MgATP 2, MgCl_2_ 2, CaCl_2_ 1 and QX-314 5 (pH adjusted to ~7.3 with CsOH).

Membrane potential values were corrected for a liquid junction potentials of ~16 mV (calculated with the Clampex software; Molecular Devices, Sunnyvale, CA, USA). The stability of the patch was checked by repetitively monitoring the input and series resistance during the experiments. Cells exhibiting 15% changes were excluded from the analysis. The series resistance was <20 MΩ and it was not compensated.

The effect of isoguvacine on cells firing was studied in cell-attached recordings. In these cases, the patch pipette was filled with ACSF. Isoguvacine (10 μM) was applied in the bath *via* the perfusion system for 60 s.

Single-channel recordings were achieved in cell-attached mode. For GABA_A_-evoked single-channel events, patch pipettes were filled with a solution containing (in mM): NaCl 120, KCl 5, tetraethylammonium-Cl 20, 4-aminopyridine 5, CaCl_2_ 0.1, MgCl_2_ 10, glucose 10, HEPES emisodium salt 10 plus GABA (3 μM). For NMDA-evoked single channel current, patch pipettes were filled with a nominally Mg^2+^-free ACSF containing NMDA (10 μM) and glycine (1 μM). The resting membrane potential (*E*_*m*_) of CA3 pyramidal neurons was estimated from the reversal potential of NMDA-induced single-channel currents measured in cell-attached configuration. The rationale behind is that NMDA currents reverse near 0 mV and therefore in cell attached they should reverse at a holding potential on the pipette *V*_*p*_ = *E*_*m*_. For single-channel recordings, the patch pipettes had a resistance of 15–20 MΩ^54^.

### Data analysis

Data were transferred to a computer hard disk after digitization with an A/D converter (Digidata 1322, Molecular Devices). Data acquisition (digitized at 20 kHz and filtered at 2 kHz) was performed with pClamp 9.2 software (Molecular Devices, Sunnyvale, CA, USA). Input resistance and cells capacitance were measured online with the membrane test feature of the pClamp software.

Spontaneous EPSCs and GPSCs were analyzed with pClamp 9 (Molecular Devices, Sunnyvale, CA, USA). This program uses a detection algorithm based on a sliding template. The template did not induce any bias in the sampling of events because it was moved along the data trace by one point at a time and was optimally scaled to fit the data at each position. All the collected events were averaged and the peak of the mean current amplitude was calculated.

The decay phase of miniature GPSCs was calculated from averaged traces by fitting it with an exponential function in the form:


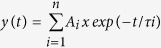


where τ_*i*_ and *A*_*i*_ are the time constants and relative fractions of respective components. Synaptic currents were usually fitted with a single exponential.

The amplitude of the tonic current was estimated by the outward shift of the baseline current after the application of the GABA_A_ receptor channel blocker picrotoxin (100 μM). Only current that exhibited a stable baseline were analyzed.

The extrapolated reversal potential of single-channel recordings was estimated by fitting the I–V curves with linear regression using Origin (Northampton, MA, USA).

To measure the effect of isoguvacine on neuronal firing, we calculated the ratio between the mean firing frequency during 5 min window preceding and 40 s window following the application of the drug.

All values are presented as mean ± SEM. Statistical comparison was performed using the Student’s unpaired *t*-test. A *p* value < 0.05 was considered as statistically significant.

### Drugs

Drugs were applied in the bath *via* a three-way tap system, by changing the superfusion solution to one differing only in its content of drug(s). Drugs used were: SR 95531 hydrobromide (gabazine), DNQX and picrotoxin purchased from Ascent Scientific (UK), isoguvacine from Sigma-Aldrich (Italy) and TTX from Latoxan (Israel). Stock solutions were made in distilled water and then aliquoted and frozen at −20 °C. DNQX was dissolved in DMSO. The final concentration of DMSO in the bathing solution was 0.1%. At this concentration, DMSO alone did not modify the membrane potential, input resistance or the firing properties of neurons.

### Modeling

All simulations were carried out using the NEURON environment (NEURON v7.4^55^). The model and simulation files will be available for public download under the ModelDB section of the Senselab database suite (http://senselab.med.yale.edu, acc.n. 188548).

The simple network used for the purposes of this work was composed of 2 interneurons and one principal cell, connected as discussed later (see Results for modeling). All cells were modeled with a single compartment, with active properties taken from a CA3 realistic model (ModelDB acc.n. 101629^56^) and from a realistic model of interneurons (ModelDB acc.n. 87546^57^). The passive properties were adapted to be consistent with the experimentally measured input resistance under control conditions (~1 GΩ). The peak conductance of all ion channels for the principal cell was manually adjusted to roughly reproduce the experimental findings under current clamp and control conditions. Synaptic inputs were implemented with a double exponential conductance change. External excitatory inputs on all neurons were implemented with AMPA-like synapses with a reversal potential of 0 mV, and τr = 0.5 msec and τd = 5 msec for the rise and decay time, respectively. They were randomly (poisson) activated at an average frequency of 50 Hz. Coordinated network activity on the CA3 cell, generating GDPs, was implemented using a synapse with a reversal potential of −40 mV, τr = 5 msec, and τd = 200 msec, randomly activated (poisson) at an average frequency of 0.3 Hz. For the synapse connecting the two interneurons (see later), we used a reversal potential of −60 mV, τr = 0.5 msec, and τd = 10 msec, whereas for the GABA-ergic synapse on the principal cell we used a reversal potential of −19 mV, τr = 0.5 msec, and τd = 10 msec. The reduced CA3 excitability suggested by experimental findings in BTBR mice, was implemented with a 52% increase of the potassium K_M_ current, which reduced the neuron’s input resistance to 0.75 GΩ (in agreement with experimental findings). The overall effects on the network were modeled with an 85% reduction of the peak inhibitory synaptic conductance of the INT1->INT2 synapse.

In implementing this model, we considered that it needed to reproduce two independent key experimental findings under BTBR conditions: 1. the reduction of intrinsic CA3 excitability in BTBR, and 2. the increased level of GABA-ergic activity. The crucial effect here was the reduction in intrinsic CA3 excitability and not the cellular mechanism(s) responsible for it, which may be an independent combination of a number of different cellular and/or subcellular changes (i.e. subthreshold sodium, calcium, and/or Ca-dependent potassium currents). However, a more specific investigation would have required a separate experimental investigation, somewhat outside the scope of this paper. For this reason, we have chosen the relatively simple and direct way to increase the K_M_, which has already been experimentally shown to be involved in changes of pyramidal cells excitability following patho-physiological conditions^58^,^59^,^60^,^61^,^62^.

## Additional Information

**How to cite this article**: Cellot, G. *et al*. Premature changes in neuronal excitability account for hippocampal network impairment and autistic-like behavior in neonatal BTBR T+tf/J mice. *Sci. Rep.* **6**, 31696; doi: 10.1038/srep31696 (2016).

## Supplementary Material

Supplementary Information
