# Corrigendum: Premature changes in neuronal excitability account for hippocampal network impairment and autistic-like behavior in neonatal BTBR T+tf/J mice

**DOI:** 10.1038/srep39726

**Published:** 2017-01-09

**Authors:** Giada Cellot, Laura Maggi, Maria Amalia Di Castro, Myriam Catalano, Rosanna Migliore, Michele Migliore, Maria Luisa Scattoni, Gemma Calamandrei, Enrico Cherubini

Scientific Reports 6: Article number: 3169610.1038/srep31696; published online: 08 16 2016; updated: 01 09 2017

In this Article, an affiliation has been omitted for Myriam Catalano. The correct affiliations are listed below:

Department of Physiology and Pharmacology, Sapienza University, Rome, Italy.

Istituto Neurologico Mediterraneo (INMED), Pozzilli, Italy.

