# Supplementary material for: Premature changes in neuronal excitability account for hippocampal network impairment and autistic-like behavior in neonatal BTBR T+tf/J mice
Source: Sci Rep. 2016 Aug 16;6:31696. doi: 10.1038/srep31696 (PMC4985660; doi:10.1038/srep31696)
Supplement: Supplementary Information [file srep31696-s1.pdf]

Supplementary Information

Premature changes in neuronal excitability account for hippocampal network impairment and autistic-like behavior in neonatal BTBR T+tf/J mice

Giada Cellot[1], Laura Maggi[2], Maria Amalia Di Castro[2], Myriam Catalano[2], Rosanna Migliore[3], Michele Migliore[3], Maria Luisa Scattoni[2], Gemma Calamandrei[2] and Enrico Cherubini[1,4*]

1. International School for Advanced Studies, via Bonomea 265, 34136 Trieste, Italy
2. Department of Physiology and Pharmacology, Sapienza University, Rome
3. Institute of Biophysics, National Research Council, 90146 Palermo, Italy
4. Department of Cell Biology and Neurosciences, Istituto Superiore di Sanità, Rome, Italy
5. European Brain Research Institute, via del Fosso di Fiorano 64, 00143 Rome, Italy

*****Corresponding author: Enrico Cherubini**,** International School for Advanced Studies (SISSA), Via Bonomea 265, 34136 Trieste, Italy**,** E-mail: cher@sissa.it

Figure S1

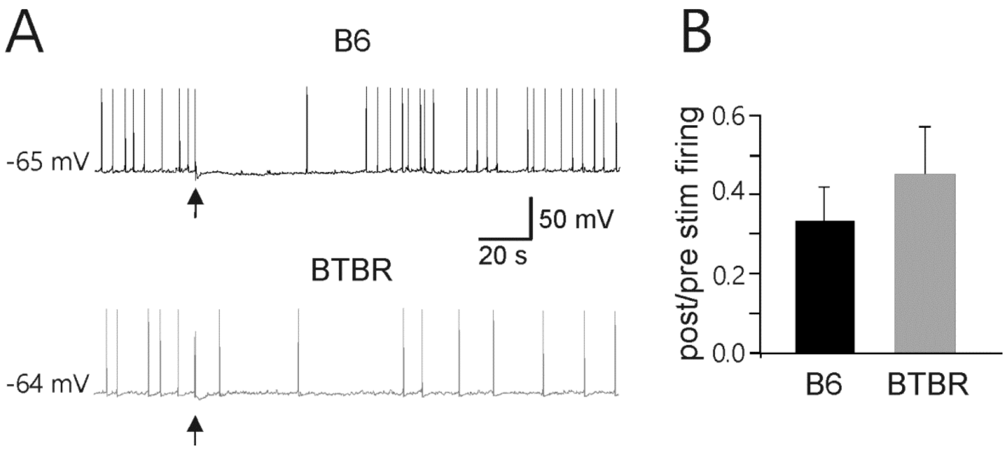

GABA released from GABAergic interneurons inhibits adult CA3 principal cells in both B6 and BTBR mice.

A. Two sample traces from adult B6 and BTBR mice showing inhibition of spontaneous firing upon stimulation (in the presence of DNQX, 10 μM; arrows) of GABAergic interneurons localized in *stratum radiatum*. B. Each column represents the ratio between the post and pre stimulus firing observed in B6 (7 cells from 3 animals, black) and BTBR mice (5 cells from 3 animals, grey). The ratio was 0.34±0.1% (p<0.01) and 0.45±0.14% (p=0.014) in B6 and BTBR mice, respectively; differences between the two genotypes were not significant (p=0.52)
